# HoloLens-Based AR System with a Robust Point Set Registration Algorithm

**DOI:** 10.3390/s19163555

**Published:** 2019-08-15

**Authors:** Jong-Chih Chien, Yao-Ren Tsai, Chieh-Tsai Wu, Jiann-Der Lee

**Affiliations:** 1 Degree Program of Digital Space and Product Design, Kainan University, Taoyuan 33857, Taiwan; 2 Department of Electrical Engineering, Chang Gung University, Taoyuan 333, Taiwan; 3 Department of Neurosurgery, Chang Gung Memorial Hospital at LinKou, Taoyuan 333, Taiwan; 4 Department of Electrical Engineering, Ming Chi University of Technology, New Taipei City 24301, Taiwan

**Keywords:** stereo vision, ICP, Helmet Mounted Display, DRWP-ICP

## Abstract

By the standard of today’s image-guided surgery (IGS) technology, in order to check and verify the progress of the surgery, the surgeons still require divert their attention from the patients occasionally to check against the display. In this paper, a mixed-reality system for medical use is proposed that combines an Intel RealSense sensor with Microsoft’s Hololens head-mounted display system, for superimposing medical data onto the physical surface of a patient, so the surgeons do not need to divert their attention from their patients. The main idea of our proposed system is to display the 3D medical images of the patients on the actual patients themselves by placing the medical images and the patients in the same coordinate space. However, the virtual medical data may contain noises and outliers, so the transformation mapping function must be able to handle these problems. The transform function in our system is performed by the use of our proposed Denoised-Resampled-Weighted-and-Perturbed-Iterative Closest Points (DRWP-ICP) algorithm, which performs denoising and removal of outliers before aligning the pre-operative medical image data points to the patient’s physical surface position before displaying the result using the Microsoft HoloLens display system. The experimental results shows that our proposed mixed-reality system using DRWP-ICP is capable of performing accurate and robust mapping despite the presence of noise and outliers.

## 1. Introduction

AR/MR (Augmented reality/mixed reality) is a field of study that is rapidly catching the interests of researchers of medical and image processing technologies [1,2]. This study is on the use of AR in the field of image-guided surgery [3], which seeks to reduce invasiveness by showing the doctor the exact locations of the lesions on the skin surface of the patient. An important part of any AR-based research is the problem of data registration; i.e., how to register two sets of data points, e.g., the image data point and the physical object’s surface data points, in the same coordinate space using alignment so as to superimpose two sets of data points accurately. The traditional method used for registration is the ICP (iterative closest points) algorithm, introduced by Besl and Zhang [4]. It uses non-elastic transformations to convert 3D data points from one coordinate system to another. The problems with it include that it requires a good starting position to start the alignment in order to get a good result, and any noise in the data could potentially corrupt the final alignment result. Later, Andriy et al. proposed the coherent point drift (CPD) [5], the main idea of which is to initialize a Gaussian mixture model and use the expectation-maximization calculation to move the centroid of the mixture. The CPD uses a uniform distribution component to account for the outliers, and could also handle some noise in the data. However, the CPD does not consider the scaling factor, so there is a possibility of deformation if there are distortions between the floating and reference datasets. The point cloud library-ICP (PCL-ICP) [6] calculates iteratively using the least squares method to obtain a rotational matrix and a displacement matrix in order to align the set of floating data points to the set of reference data points, which are assumed to be in the same coordinate system. The CloudCompare-ICP (CC-ICP) [7] implements the greedy four points congruent sets registration methods for coarse alignment then uses the standard ICP and rigid transformations for refinement. Recently, Wu [8] presented an improved ICP algorithm, the Improved-ICP, that uses weighting and perturbation to match a single floating data set to the reference data set, but it does not consider the problem of noise and outliers. Generally, the traditional ICP algorithm is considered fine to use for general purposes. However, the traditional ICP has multiple issues: (1) If there are gaps in the floating data that do not correspond well to the reference data, then a good match may not be achievable; (2) If the alignment process is started from a random starting position, then it could result in a locally optimal result, so a global optimal solution is not guaranteed; even the Globally optimal-ICP algorithm [9], which claimed that it can achieve global optimal solutions, was shown in Wu’s paper [8] to be unable to reach better solutions without performing initial coarse alignments; and (3) Outlier data points or noise in the original floating data points may affect the final result of the alignment.

In this paper, we introduce the DRWP-ICP (Denoised-Resampled-Weighted-and-Perturbed ICP), which has the advantages the other ICP-based method do not. It seeks to improve the traditional ICP algorithm by preprocessing the floating datasets before alignment, in order to achieve a smoother, less noisy and more uniform floating dataset. For each floating dataset, it first performs denoising (**D**) to remove noisy data and outliers, then resample (**R**) the floating datasets set which results in a better, smoother floating data set. It then performs line-search with proper weights (**W**) in the floating data in order to find matching reference data, and uses perturbation (**P**) to escape from local minima if the searching result is not below a small preset threshold in order to reach a better, near optimal, solution. It will be shown in the results of comparison experiments in a later section that the DRWP-ICP outperformed other ICP-based algorithms with greater accuracy and shorter running times. So, the DRWP-ICP should result in a better match of the floating data sets to the reference data set more quickly. It can be used in a medical AR system using HoloLens [10] and allows the surgeon to see his patient at the same time as the pre-operatively obtained medical data by accurately superimposing them, and allows the surgeon to focus his/her attention more on the patient during an image-guided surgery (IGS) operation.

## 2. Method

**Figure 1:**
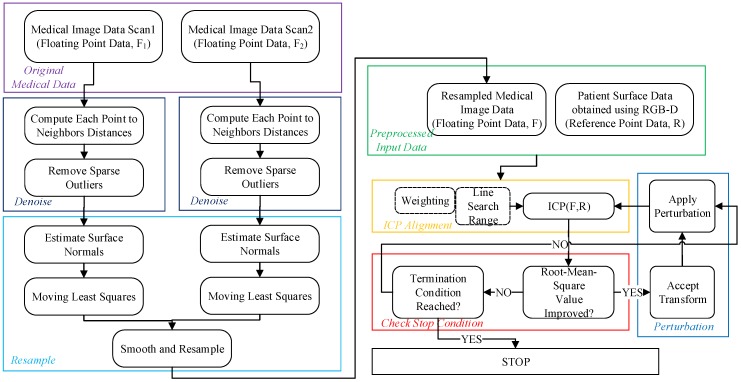
The flowchart of the Denoised-Resampled-Weighted-and-Perturbed-Iterative Closest Points (DRWP-ICP) algorithm.

A dummy head was used to simulate the patient’s head. A set of CT scans using Intel RealSense [11] sensor were performed on the dummy head in order to obtain a stack of images which were processed to build a 3D model of the dummy head, these constituted the reference set data, R. The Intel RealSense RGB-Depth sensor was used to obtain the patient’s surface data points, and Point Cloud Library was used to reconstruct the 3D information of the patient’s surface as the floating data point set F_1_. This process was repeated again to obtain a second floating points set, F_2_. So we had the reference dataset, R, and the floating data point sets, F_1_ and F_2_. The DRWP-ICP algorithm first performed preprocessing of the floating datasets by denoising then resampling to achieve a more accurate floating dataset, F. Then the DRWP-ICP performed a weighted line search scheme [12] to match each point in the floating dataset to the reference dataset. The purpose of the weighting, which was proportional to the distance of the corresponding points in the floating dataset and the target dataset, was to prevent the accumulation of errors when multiple floating points match the same target point. However, a weighted line search could still result in reaching local minima rather than the global minimum, so a perturbation scheme was added to escape from the local minima. Once the floating dataset was matched to the reference dataset, the resulting data points were displayed on the doctor’s HoloLens headwear by aligning and overlaying the virtual data to the actual patient’s skin surface. The flowchart of the DRWP-ICP is shown in Figure 1 below.

### The DRWP-ICP Procedure

Step 1. Data Input. The pre-operative medical/CT image was the set of reference points R, and the patient’s surface data captured by the RGB-Depth sensor was the set of floating points, F_1_ and F_2_.

Step 2. Floating Data Processing by Denoising and Resampling. The main idea in the denoising stage was to use a statistical filter on the neighborhood of each data point and remove points that did not meet certain criteria. The sparse outlier removal method was based on the calculation of the distance distribution from the point in the input data to the adjacent point. The statistical filter assumed that the average distance between all points in the point cloud and the nearest K neighbors satisfies the Gaussian distribution. According to the mean variance, a distance threshold can be determined. When the average distance between a point and the nearest K points was greater than this the threshold, the points beyond this threshold were then removed. First the average distance between each point and its nearest K neighbors was calculated, and then the mean μ and standard deviation σ of all the average distances were calculated. The distance threshold can be defined as:

d_max_ = μ + α × σ, (1)

where α is a constant known as the scale factor and its values depend on the number of neighbors. Finally each point whose distance was greater than d_max_ was removed. In the resampling stage, the main idea was to use a moving least squares surface reconstruction method [13] to smooth and resample the noisy data. This algorithm attempted to reconstruct the missing portion of the surface by high-order polynomial interpolation between surrounding data points. First, the corresponding surface was obtained from the acquired point cloud data set, and then the surface normal was calculated from the model. The solution for estimating the surface normal was simplified by analyzing the eigenvectors and eigenvalues (or PCA—principal component analysis [14]) of the covariance matrix from the nearest neighbor of the query point. The purpose of the surface normal estimation was to first obtain the nearest neighbor element of point p and then calculate the surface normal n of point p. Then we checked whether the direction of n was consistently pointing to the viewpoint. If not, it was flipped. The viewpoint coordinates were preset to (0,0,0). The least squares method is a global method, which cannot meet the requirements for localized processing, and it can cause difficulty in model setting and computational instability for a large number of point data. Therefore, this method used the moving least squares method to solve the registration problem. The scatter adaptability also has the advantages of local curve fitting, being adaptable to distributed difference characteristics, as well as having a high precision. The moving least squares method divided the region into meshes, then looped through each point in the mesh to compute the shape function at each node, then finally connected the nodes to form a fitted curve surface. This was done for both floating datasets, then the data were resampled from both datasets to generate a smoother, more accurate floating dataset, F.

Step 3. ICP Alignment. For any given point f in F, we found its nearest corresponding point r in R by calculating the minimum distance between f and each possible r, d. Then weight values were assigned to each pair in the same group of data that matches the same reference point, then the min value was calculated according to the following:

(2) $$\alpha_{min}=\frac{1}{N_{B}-N_{C}}\sum_{i=1}^{N_{B}-N_{C}}d_{i}$$

where *N_B_* is the total number of points in the floating data set, and *N_C_* is the number of points to be excluded. This number was determined after sorting *d_i_* in order to reduce the overall complexity. Then the weight was assigned as follows:

(3) $$w=\{\begin{array}{c} 1\\ \frac{\alpha_{mean}}{{\left(d_{i}\right)}^{t}} \end{array}\;\begin{array}{c} if\;d_{i}<\alpha_{mean}\\ else \end{array}.$$

After the assignments were completed, then the objective function which seeks to minimize the root mean square (RMS) value, was calculated. The way to evaluate the objective function was similar to gradient descent, so in order to speed up the search, the line search method was implemented, which searched in the nonlinear space of the objective function, F, in order to arrive at a minimum faster. If the solution was accepted, then a transformation matrix T’ was obtained by the ICP algorithm, and if the RMS value calculated was less than the previous iteration, then T’ replaced the currently optimal transformation matrix T, then automatically jumped to the perturbation stage. Otherwise, whether the stop condition had been reached had to be determined first.

Step 4. Stop Condition. The stop condition was so that if the RMS value is less than a preset threshold, or if the number of iterations so far was greater than a preset value, then the stop condition was determined to be reached, and the algorithm stopped.

Step 5. Perturbation. If we let T_init_ be the initialization transformation T before the ICP begins and let the T_temp_ be the transformation matrix for the converged solution, then the range to explore for T_temp_ would be in the range of r, where

r = | T_init_ − T_temp_ | (4)

since T_init_ and T_temp_ are both 4 × 4 transformation matrices, the range of r represents the range of possible discrete 4 × 4 transforms. Now suppose T_temp_ only reached a local minimum, then the transformation for perturbation was chosen from the range of r, where probability of being selected was:

(5) $$p\left(\theta \right)=\{\begin{array}{c} \alpha \theta^{2}\\ 0 \end{array}\;\begin{array}{c} if\;-\alpha r<\theta <+\alpha r\\ otherwise \end{array}$$

where α is the scaling factor to help expand the perturbation range if the current search range was not sufficient to escape from a local solution.

**Figure 2:**
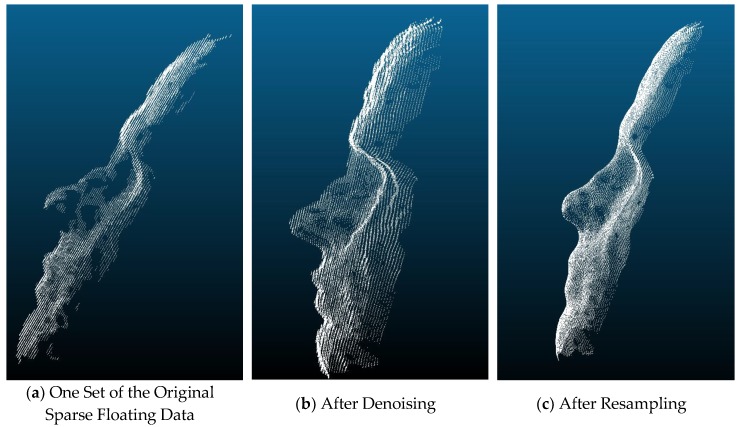
Simulated effects of denoising and resampling of the original floating data.

The illustrations for the effects of denoising and resampling can be seen below in Figure 2.

## 3. Experimental Results

In the experiments, a specially-designed marker was placed next to the patient’s head (dummy head), so when the camera detected the marker plate, the built-in Vuforia image tracking SDK (Software Development Kit) [15] would know the spatial position of the patient’s head so as to map the HoloLens’ coordinate system to the marker’s coordinate system, then the medical image data points were aligned to the patient’s surface information via different ICP-based algorithms, including our proposed method. The RMS (root-mean-squared) errors and alignment times were recorded as the performance of DRWP-ICP was compared to other well-known ICP-based algorithms proposed in the literature. The final results were then displayed in the HoloLens output. In this setup, each floating dataset was composed of 48,596 points.

**Figure 3:**
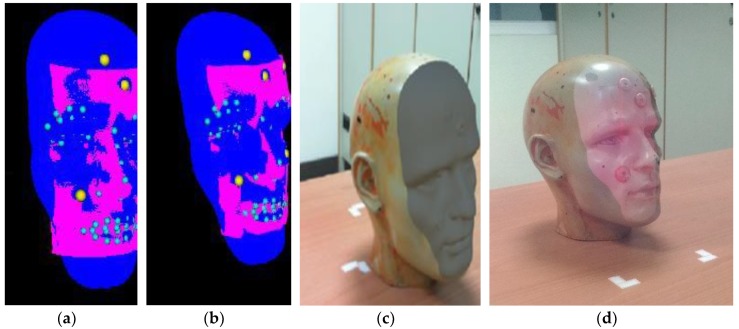
Sample alignment results. (**a**) Improved-ICP; (**b**) DRWP-ICP; (**c**) without HoloLens; and (**d**) with HoloLens.

The hardware setup included a desktop running Windows with Intel^®^ Core™ i5-4460 CPU, an Intel^®^ RealSense RGB-Depth sensor (Santa Clara, CA, USA), and the Microsoft HoloLens MR system (Redmond, WA, USA). The software and user interface was developed using Unity [16] and OpenCV [17]. In order to compare the DRWP-ICP’s accuracy with other ICP-based algorithms, positional values of five references points were first obtained by using the MicroScribe G2X digitizer coordinate tracking device [18]. The number of pre-measured reference points was chosen based on the experimental setup in the Improved-ICP paper [8]. Figure 3 shows possible alignment results, where Figure 3a is one of the alignment results through the use of the Improved-ICP algorithm (blue points represent the reference dataset, the pink points represent the floating dataset (that are different from the values of the reference dataset), and yellow points represent the five reference points whose positional values are pre-measured); Figure 3b is one of the alignment results through the use of our proposed DRWP-ICP algorithm (the blue points represent the reference dataset, the pink points represent the floating dataset (that are different from the values of the reference dataset), and yellow dots represents the five pre-measured reference points); and Figure 3c is the output taken using a regular camcorder, without Hololens, with the areas representing floating data points marked in gray; and Figure 3d is the outputs on the Hololens display, with the reference data points (in pink) overlaying the dummy. As it can be seen, HoloLens provided an augmented display over the reality of the dummy head.

The experiments were designed for two groups of tests, identical except for the pre-alignment process. In the first group, no initial coarse pre-alignment was performed for each ICP algorithm under testing for their effectiveness under non-ideal conditions. In the second group of experiments, initial coarse pre-alignments were performed for each ICP algorithm (except Improved-ICP and DRWP-ICP algorithms) before further testing. Each of these groups were divided into two subgroups of experiments. Each of the subgroups had several sets of experiments to be performed: The first set of experiments tested the efficacy of each ICP-based algorithm in the presence of sparse floating data. The second set of experiments tested the efficacy of each ICP-based algorithm in the presence of additive Gaussian noises. The third and final set of experiment tested the efficacy of each ICP-based algorithm in the presence of outliers. Both averaged alignment time (excluding coarse alignment), and the average RMS errors of five pre-measured reference points were recorded after multiple repeated experiments. The ICP-based algorithms to be tested included the PCL-ICP, the CPD, the CC-ICP, the CC-ICP with its denoise function enabled (CC-ICP+D), the Improved-ICP, and our proposed DRWP-ICP.

### 3.1. Without Coarse Alignment (Random Staring Position)

#### 3.1.1. Sparse Data

**Figure 4:**
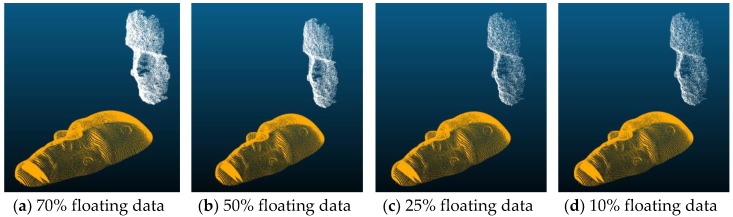
Examples of sparse floating (white) and reference points (gold) used in the experiments.

In order to test the effect of sparse data, the cases included testing 100% of floating data used in the alignment as reference, as well when only 70%, 50%, 25%, and 10% of the floating data points were used. The searches all started from random positions, including random distance and orientation. The following figure, Figure 4, shows examples of the floating data points in white and the reference data points in gold. Table 1 and Table 2 show the average, maximum and minimum errors; and alignment times, respectively. Since the alignment times for CC-ICP and CC-ICP+D were almost the same, no extra column was used for the CC-ICP+D alignment times.

#### 3.1.2. Noise

**Figure 5:**
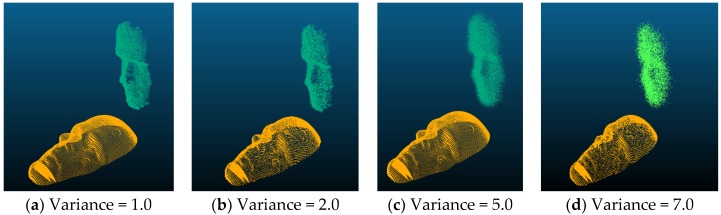
Examples of floating (green) with Gaussian noises and reference points (gold) used in the experiments.

In this set of experiments, Gaussian noises of different variances, i.e., 1.0, 2.0, 5.0, and 7.0, were added to the floating datasets. The following figure, Figure 5, shows examples of the floating data points in green and the reference data points in gold. Table 3 and Table 4 show the average, minimum, maximum errors; and the alignment times, respectively.

#### 3.1.3. Outliers

**Figure 6:**
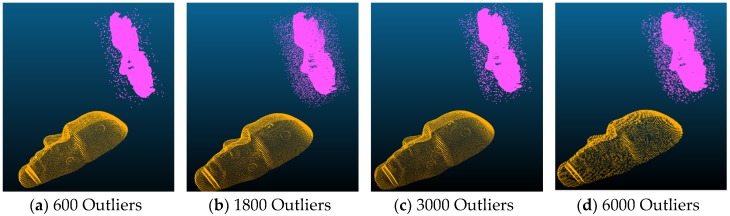
Examples of floating (pink) with Gaussian noises and reference points (gold) used in the experiments.

In this set of experiments, different numbers of outliers, i.e., 600, 1800, 3000, and 6000, were deliberately added to the floating dataset. The following figure, Figure 6, shows examples of the floating data points in pink and the reference data points in gold. Table 5 and Table 6 show the average, minimum, maximum errors; and alignment times, respectively.

### 3.2. With SAC-IA Coarse Pre-Alignment

**Figure 7:**
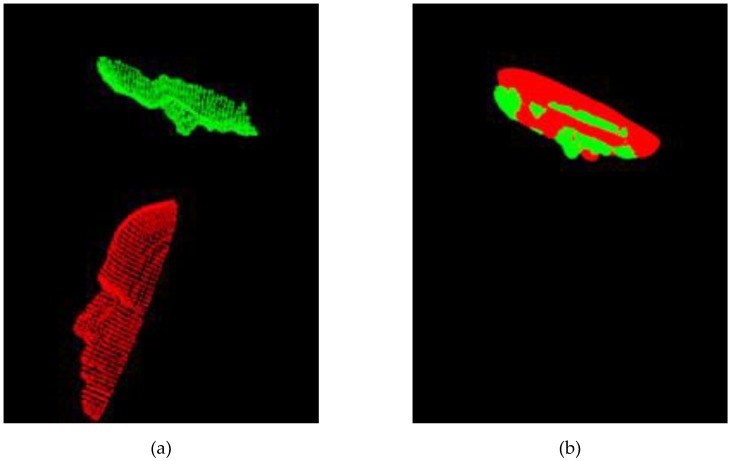
A possible scenario of (**a**) pre-coarse-alignment (**b**) post-coarse-alignment positions.

In this subgroup of experiment a preliminary coarse pre-alignment, the sampling consensus initial alignment (SAC-IA) [19], was first performed for each ICP-based algorithm. However, no coarse alignments were performed for the Improved-ICP algorithm, because its authors indicated no coarse alignment is necessary. Also, no coarse alignment was performed for our proposed DRWP-ICP, because we also believe that it is not necessary. Their values in the tables below are exactly the same as their values in the first group of experiments and are listed for reference purposes only. The figure below, Figure 7, illustrates the process of coarse alignment, where the green points represents the floating data and the red points represent the reference data. Table 7 displays the average RMS errors after the coarse alignment for the five reference points.

#### 3.2.1. Sparse

Again, in order to test the effects of different ICP-based algorithm in the presence of sparse data, only 70%, 50%, 25%, and 10% of the floating data points were used. The original 100% of the data was also used for reference purposes. Table 8 and Table 9 show the average, minimum, maximum errors; and alignment times, respectively (not including the times taken for coarse pre-alignments).

#### 3.2.2. Noise

In this set of experiments, Gaussian noises of different variances, i.e., 1.0, 2.0, 5.0, and 7.0, were added to the floating datasets. The mathematical expression of Gaussian distribution is:

(6) $$ƒ\left(x\right)=\frac{1}{\sqrt[{\sigma }]{2\pi }}e^{-\frac{{\left(x-µ\right)}^{2}}{2\sigma^{2}}}$$

where μ is the sample mean and the σ is the sample standard deviation. Table 10 and Table 11 show the average, minimum, maximum errors; and alignment times, respectively.

#### 3.2.3. Outliers

In this set of experiments, different numbers of outliers, i.e., 600, 1800, 3000, and 6000, were deliberately added to the floating dataset. Table 12 and Table 13 show the average, minimum, maximum errors; and alignment times, respectively.

A final experiment using floating datasets with sparse data (10%), Gaussian noise (Variance = 7.0), and outliers (6000) was tested in order to test the robustness of our proposed DRWP-ICP when all three conditions exist. Table 14 below shows the average, maximum, and minimum alignment errors of the five registration methods. Coarse alignments were performed for PCL-ICP, CPD, and CC-ICP.

## 4. Discussion

The same observation as the Improved-ICP paper [8] can be drawn from the differences between the RMS values of experiments with and without coarse alignment: that the performances of the PCL-ICP, CPD and CC-ICP algorithms can all be improved by a coarse pre-alignment. The results from the experiments without alignments, seem to partially validate the claims by CPD, that is more robust under noisy and outliers, because all of the RMS errors of CPD were less than those of the PCL-ICP and the CC-ICP. However, in the experiments with coarse alignment, the performance of CPD dropped below the other ICP-base methods when that data became too noisy, or had too many outliers. Also in all the experiments the CPD took longer to terminate. Also, an obvious observation can also be made: noisier data, or data that have more outliers, will take longer to align. In the case of sparse data, the sparser the data, the less time it takes to perform alignment. Another interesting observation concerns the effects of the additional coarse pre-alignment for PCL-ICP, CPD, and CC-ICP algorithms. For example, by first performing coarse alignment, the alignment time for the PCL-ICP, CPD, and CC-ICP algorithms took shorter times to achieve their respective, and better alignment results, including sparse, noisy, and in most of the outliers cases. We believe that this is due to the fact that CPD and the other ICP-based methods, except Improved-ICP, did not fully consider being trapped into local minima, so when the floating data started from a random starting position, they were trapped into sub-optimal solutions. Our study proposes a method to escape from local minima even when the search appears to have converged. The coarse alignment reduced the search-space for the other methods and allowing them to reach better solutions.

Another observation is that by including the pre-alignment operation, and without counting the time for the pre-alignment, most of the alignment times for the PCL-ICP, CPD, and CC-ICP algorithms actually decreased, even though they achieved better results with pre-alignments. This is an interesting phenomena, however, because their performances still lag behind DRWP-ICP, it remains just a mildly interesting phenomena.

The entries in the tables above show that in almost all of the test cases, including RMS errors and running times, the proposed DRWP-ICP exhibited much better performances than the other algorithms in the test group, even the Improved-ICP algorithm. However, it is also observed that the CC-ICP with denoise function turned on can perform better. This is even more obvious with our coarse alignment, CC-ICP with denoise function was able to also reach near optimal solutions. This shows the importance of a good coarse alignment for the CC-ICP algorithm. So, these results combined show that the operations of denoising and resampling do affect the final results of alignment, and implies that DRWP-ICP algorithm is not only more accurate but also more robust than the other algorithms tested in the experiment for point set registration/alignment. This observation is valid since the original coarse alignment algorithm included in CC-ICP was not sufficient for it to reach near optimal solutions. The results in Table 14 show that it is superior even when all three conditions exists; i.e., noisy, sparse, and having outliers. It is also clear that the DRWP-ICP algorithm also does not require pre-alignment in order to reach near globally optimal solutions, so there is no need to consider pre-alignment in order to improve DRWP-ICP, which is makes it more efficient than the other algorithms that require pre-alignments in order to achieve better results.

## 5. Conclusions

In this work, a mixed-reality system for aiding the surgeons during image-guided surgeries is proposed. The pre-operative medical images are first taken and then superimposed on the patient through the use of the DRWP-ICP algorithm, which was shown by experiments to be superior both in performance and robustness when compared to other ICP-based algorithms in the presence of sparse data, noisy data, outlier data, or a combination of them. The aligned results are then displayed on the Microsoft’s HoloLens display as the medical data are superimposed on the patient. The use of the proposed MR system implies that the surgeon does not need to remove his focus from the patient during the operation in order to ascertain the progress of the operation, and that the surgeon can have full confidence that the medical data will be accurately displayed over the patient.

## Figures and Tables

**Table 1:** The Average, Minimum and Maximum of Reference Points’ Alignment Errors.

**Average Root Mean Square (RMS) Errors of Five Reference Points of Different ICP-based Methods (mm)**						
**Method/Sparsity**	**DRWP-ICP**	**Improved-ICP**	**PCL-ICP**	**CPD**	**CC-ICP**	**CC-ICP+D**
100% (~48,596)	0.932	2.248	46.948	15.494	33.562	7.88
70% (~34,017)	1.308	3.314	46.954	15.58	49.52	7.93
50% (~242,980	1.512	3.444	46.948	15.494	49.5	7.965
25% (~12,149)	1.806	3.262	46.920	15.656	49.516	8.156
10% (~4860)	1.82	3.644	47.026	15.46	49.502	8.04
**Minimum of RMS Errors of Five Reference Points (mm)**						
**Method/Sparsity**	**DRWP-ICP**	**Improved-ICP**	**PCL-ICP**	**CPD**	**CC-ICP**	**CC-ICP+D**
100% (~48,596)	0.37	1.96	24.21	6.89	16.48	5.05
70% (~34,017)	0.77	2.61	24.13	6.86	25.09	5.04
50% (~242,980	1.07	2.89	24.26	6.97	25.03	5.1
25% (~12,149)	1.44	2.79	24.33	7.16	24.99	5.23
10% (~4860)	1.36	3.16	24.03	6.94	24.77	5.04
**Maximum of RMS Errors of Five Reference Points (mm)**						
**Method/Sparsity**	**DRWP-ICP**	**Improved-ICP**	**PCL-ICP**	**CPD**	**CC-ICP**	**CC-ICP+D**
100% (~48,596)	1.49	2.84	66.45	22.63	60.05	10.44
70% (~34,017)	1.77	3.74	66.54	22.59	75.77	10.56
50% (~242,980	2.15	4.22	66.43	22.53	75.71	10.55
25% (~12,149)	2.29	3.75	66.32	22.69	75.75	10.81
10% (~4860)	2.51	3.99	66.73	22.77	75.98	10.71

**Table 2:** Alignment times (seconds).

Method/Sparsity	DRWP-ICP	Improved-ICP	PCL-ICP	CPD	CC-ICPs
100% (~48,596)	3.85	6.21	80	184	10
70% (~34,017)	3.16	5.97	81	164	9
50% (~242,980	3.01	5.1	77	162	8
25% (~12,149)	2.99	4.76	65	160	5.4
10% (~4860)	2.54	4.65	49	145	4.1

**Table 3:** The Average, Minimum and Maximum of Reference Points’ Alignment Errors.

**Average RMS Errors of Five Reference Points of Different ICP-based Methods (mm)**						
**Method/Noise**	**DRWP-ICP**	**Improved-ICP**	**PCL-ICP**	**CPD**	**CC-ICP**	**CC-ICP+D**
Variance = 1.0	1.024	2.018	46.962	15.492	49.536	12.285
Variance = 2.0	1.048	2.114	46.902	15.492	49.4	9.534
Variance = 5.0	1.49	2.398	46.764	15.536	48.874	30.5
Variance = 7.0	1.662	2.644	46.61	15.536	48.538	31.35
**Minimum of RMS Errors of Five Reference Points (mm)**						
**Method/Noise**	**DRWP-ICP**	**Improved-ICP**	**PCL-ICP**	**CPD**	**CC-ICP**	**CC-ICP+D**
Variance = 1.0	0.4	1.38	24.24	6.93	24.07	7.34
Variance = 2.0	0.43	1.42	24.28	6.95	25.00	5.74
Variance = 5.0	1.03	1.51	24.13	7.00	24.67	20.13
Variance = 7.0	1.1	1.49	23.58	7.02	23.90	21.2
**Maximum of RMS Errors of Five Reference Points (mm)**						
**Method/Noise**	**DRWP-ICP**	**Improved-ICP**	**PCL-ICP**	**CPD**	**CC-ICP**	**CC-ICP+D**
Variance = 1.0	1.3	2.34	66.45	22.59	75.77	16.46
Variance = 2.0	1.5	2.41	66.4	22.53	75.71	12.86
Variance = 5.0	2.13	2.71	66.59	22.69	75.75	39.02
Variance = 7.0	2.49	3.27	67.01	22.77	75.98	39.94

**Table 4:** Alignment times (seconds).

Method/Noise	DRWP-ICP	Improved-ICP	PCL-ICP	CPD	CC-ICPs
Variance = 1.0	4.34	4.14	169	360	20
Variance = 2.0	6.24	8.16	231	691	26
Variance = 5.0	9.24	14.74	308	782	35
Variance = 7.0	14.65	19.89	384	1440	35

**Table 5:** The Average, Minimum and Maximum of Reference Points’ Alignment Errors.

**Average RMS Errors of Five Reference Points of Different ICP-based Methods (mm)**						
**Method/Outliers**	**DRWP-ICP**	**Improved-ICP**	**PCL-ICP**	**CPD**	**CC-ICP**	**CC-ICP+D**
600	0.992	2.232	46.902	17.794	49.472	11.036
1800	1.018	3.856	46.836	15.572	49.328	9.338
3000	1.536	4.784	46.796	15.566	49.072	27.32
6000	1.55	4.832	46.474	15.594	48.686	10.454
**Minimum RMS Errors of Five Reference Points (mm)**						
**Method/Outliers**	**DRWP-ICP**	**Improved-ICP**	**PCL-ICP**	**CPD**	**CC-ICP**	**CC-ICP+D**
600	0.37	1.71	24.19	13.81	25.0	7.22
1800	0.36	3.52	24.19	7.02	24.96	6.92
3000	1.03	3.45	24.29	7.07	24.83	16.4
6000	1.03	3.43	24.18	7.15	24.48	5.98
**Maximum RMS Errors of Five Reference Points (mm)**						
**Method/Outliers**	**DRWP-ICP**	**Improved-ICP**	**PCL-ICP**	**CPD**	**CC-ICP**	**CC-ICP+D**
600	1.35	2.61	66.47	22.26	75.7	14.56
1800	1.51	5.91	66.52	22.69	75.54	12.33
3000	2.13	5.63	66.57	22.71	75.21	39.8
6000	2.16	5.8	66.37	22.74	74.82	14.49

**Table 6:** Alignment times (seconds).

Method/Outliers	DRWP-ICP	Improved-ICP	PCL-ICP	CPD	CC-ICPs
600	5.17	35.24	248	318	12
1800	6.1	36.24	288	399	26
3000	7.17	38	352	581	33
6000	13.41	45.34	395	602	37

**Table 7:** Average RMS errors of five reference points after coarse-Alignment for original data (mm).

Reference Point	Point 1	Point 2	Point 3	Point 4	Point 5	Average of Five Points
Average Error	17.13	14.42	16.80	13.78	15.82	15.59

**Table 8:** The Average, Minimum and Maximum of Reference Points’ Alignment Errors.

**Average RMS Errors of Five Reference Points of Different ICP-based Methods (mm)**						
**Method/Sparsity**	**DRWP-ICP**	**Improved-ICP**	**PCL-ICP**	**CPD**	**CC-ICP**	**CC-ICP+D**
100% (~48,596)	0.932	2.248	1.832	2.134	7.47	0.904
70% (~34,017)	1.308	3.314	1.9	2.376	7.556	0.928
50% (~242,980	1.512	3.444	2.262	2.406	7.588	0.852
25% (~12,149)	1.806	3.262	4.338	7.742	7.824	0.822
10% (~4860)	1.82	3.644	9.284	8.28	11.838	0.904
**Minimum RMS Errors of Five Reference Points (mm)**						
**Method/Sparsity**	**DRWP-ICP**	**Improved-ICP**	**PCL-ICP**	**CPD**	**CC-ICP**	**CC-ICP+D**
100% (~48,596)	0.37	1.96	1.04	1.34	4.86	0.23
70% (~34,017)	0.77	2.61	1.12	2.16	4.86	0.24
50% (~242,980	1.07	2.89	1.71	1.61	4.93	0.28
25% (~12,149)	1.44	2.79	3.3	3.88	5.09	0.26
10% (~4860)	1.36	3.16	4.45	4.54	6.68	0.21
**Maximum RMS Errors of Five Reference Points (mm)**						
**Method/Sparsity**	**DRWP-ICP**	**Improved-ICP**	**PCL-ICP**	**CPD**	**CC-ICP**	**CC-ICP+D**
100% (~48,596)	1.49	2.84	2.29	3.01	9.54	1.27
70% (~34,017)	1.77	3.74	2.32	2.53	9.99	1.27
50% (~242,980	2.15	4.22	2.69	2.95	9.99	1.26
25% (~12,149)	2.29	3.75	5.74	10.75	10.3	1.29
10% (~4860)	2.51	3.99	13.61	11.01	15.94	1.22

**Table 9:** Alignment times (seconds).

Method/Sparsity	DRWP-ICP	Improved-ICP	PCL-ICP	CPD	CC-ICPs
100% (~48,596)	3.85	6.21	65	175	5
70% (~34,017)	3.16	5.97	64	159	5.8
50% (~242,980	3.01	5.1	61	154	5.5
25% (~12,149)	2.99	4.76	57	151	4.8
10% (~4860)	2.54	4.65	49	145	3.9

**Table 10:** The Average, Minimum and Maximum of Reference Points’ Alignment Errors.

**Average RMS Errors of Five Reference Points of Different ICP-based Methods (mm)**						
**Method/Noise**	**DRWP-ICP**	**Improved-ICP**	**PCL-ICP**	**CPD**	**CC-ICP**	**CC-ICP+D**
Variance = 1.0	1.024	2.018	1.96	3.086	8.056	0.916
Variance = 2.0	1.048	2.114	9.38	8.278	8.248	0.844
Variance = 5.0	1.49	2.398	9.436	10.16	9.666	1.12
Variance = 7.0	1.662	2.644	9.642	11.272	10.684	1.526
**Minimum RMS Errors of Five Reference Points(mm)**						
**Method/Noise**	**DRWP-ICP**	**Improved-ICP**	**PCL-ICP**	**CPD**	**CC-ICP**	**CC-ICP+D**
Variance = 1.0	0.4	1.38	1.25	2.36	5.31	0.16
Variance = 2.0	0.43	1.42	3.94	4.42	5.42	0.49
Variance = 5.0	1.03	1.51	4.65	6.48	6.76	0.71
Variance = 7.0	1.1	1.49	3.29	7.35	8.08	1.2
**Maximum RMS Errors of Five Reference Points (mm)**						
**Method/Noise**	**DRWP-ICP**	**Improved-ICP**	**PCL-ICP**	**CPD**	**CC-ICP**	**CC-ICP+D**
Variance = 1.0	1.3	2.34	2.46	5.14	10.54	1.37
Variance = 2.0	1.5	2.41	13.88	10.84	10.78	1.05
Variance = 5.0	2.13	2.71	13.78	13.03	12.51	1.63
Variance = 7.0	2.49	3.27	14.47	14.69	13.37	1.98

**Table 11:** Alignment times (seconds).

Method/Noise	DRWP-ICP	Improved-ICP	PCL-ICP	CPD	CC-ICPs
Variance = 1.0	4.34	4.14	108	240	7
Variance = 2.0	6.24	8.16	198	480	7
Variance = 5.0	9.24	14.74	216	600	15
Variance = 7.0	14.65	18.89	247	1200	32

**Table 12:** The Average, Minimum and Maximum of Reference Points’ Alignment Errors.

**Average RMS Errors of Five Reference Points of Different ICP-based Methods (mm)**						
**Method/Outliers**	**DRWP-ICP**	**Improved-ICP**	**PCL-ICP**	**CPD**	**CC-ICP**	**CC-ICP+D**
600	0.992	2.232	9.344	7.448	5.81	0.872
1800	1.018	3.856	9.336	19.878	9.478	0.874
3000	1.536	4.784	9.43	20.18	12.472	0.844
6000	1.55	4.832	10.038	20.194	14.028	0.848
**Minimum RMS Errors of Five Reference Points (mm)**						
**Method/Outliers**	**DRWP-ICP**	**Improved-ICP**	**PCL-ICP**	**CPD**	**CC-ICP**	**CC-ICP+D**
600	0.37	1.71	4.64	3.29	4.3	0.19
1800	0.36	3.52	4.71	1.48	8.14	0.23
3000	1.03	3.45	4.63	3.51	9.1	0.28
6000	1.03	3.43	3.2	1.7	10.62	0.2
**Maximum RMS Errors of Five Reference Points (mm)**						
**Method/Outliers**	**DRWP-ICP**	**Improved-ICP**	**PCL-ICP**	**CPD**	**CC-ICP**	**CC-ICP+D**
600	1.35	2.61	13.68	11.78	7.27	1.38
1800	1.51	5.91	13.64	32.53	11.33	1.31
3000	2.13	5.63	13.8	30.48	17.14	1.34
6000	2.16	5.8	15.15	33.2	18.32	1.38

**Table 13:** Alignment times (seconds).

Method/Outliers	DRWP-ICP	Improved-ICP	PCL-ICP	CPD	CC-ICPs
600	5.17	35.24	194	215	11
1800	6.1	36.24	173	384	11
3000	7.17	38	181	391	13
6000	13.41	45.34	220	461	15

**Table 14:** RMS errors of five reference points under all sparse, noisy, and outlier conditions (mm).

Method/Statistic	DRWP-ICP	Improved-ICP	PCL-ICP	CPD	CC-ICP
Average	3.678	6.976	9.807	13.034	9.193
Minimum	3.57	6.49	6.94	7.74	8.05
Maximum	3.85	7.32	13.67	19.36	10.79
